# DNA damage-induced nuclear STING translocation orchestrates innate immune activation and chromatin remodeling

**DOI:** 10.1016/j.gendis.2025.101851

**Published:** 2025-09-09

**Authors:** Yihao Wang, Zan Shen, Lingjie Li, Jianfeng Shen

**Affiliations:** a Department of Ophthalmology, Ninth People's Hospital, Shanghai Jiao Tong University School of Medicine, Shanghai Key Laboratory of Orbital Diseases and Ocular Oncology, Shanghai 200025, China; b Institute of Translational Medicine, National Facility for Translational Medicine, Shanghai Jiao Tong University, Shanghai 200240, China; c Department of Histoembryology, Genetics and Developmental Biology, Shanghai Key Laboratory of Reproductive Medicine, Key Laboratory of Cell Differentiation and Apoptosis of Chinese Ministry of Education, Shanghai Jiao Tong University School of Medicine, Shanghai 200025, China; d Department of Oncology, Sixth People's Hospital, Shanghai Jiao Tong University School of Medicine, Shanghai 200233, China

Stimulator of interferon gene (STING), a key innate immune adapter, senses cytosolic DNA to trigger type I interferon responses and is linked to cancer and autoimmune disorders.^1^^,^^2^ Beyond canonical signaling, STING acts as a proton channel promoting non-canonical autophagy and inflammation,^3^ while its nuclear translocation may facilitate DNA repair.^4^ Dixon et al identified STING redistributing from inner to outer nuclear membranes upon dsDNA or dsRNA stimulation.^5^ Nevertheless, the potential role of nuclear STING remains largely unexplored. In our study, we found that poly(ADP-ribose) polymerase (PARP) inhibitor-induced DNA damage triggered STING nuclear translocation in cancer cells. Chromatin immunoprecipitation sequencing (ChIP-seq) identified nuclear STING targets linked to chromatin remodeling and enriched transcriptional motifs. RNA sequencing (RNA-seq) data from STING wild-type and knockout cell lines confirmed nuclear STING's necessity for innate immune activation and chromatin remodeling. Our findings demonstrate that STING undergoes nuclear translocation, where it alters the immune response and chromatin remodeling of cancer cells. These results reveal novel molecular targets and non-canonical functions of nuclear STING, providing a framework for developing targeted cancer therapies to modulate STING activity.

After treating HeLa cells with BMN673, a potent PARP1/2 inhibitor, for 24 h, we observed a dose-dependent decrease in cytoplasmic STING protein levels and a significant increase in the nucleus, indicating STING nuclear translocation in response to DNA damage (Fig. 1A). Similar results were obtained with another PARP inhibitor, olaparib (Fig. 1B). Notably, unlike STING, cyclic GMP-AMP synthase (cGAS) protein levels remained unchanged, suggesting that STING nuclear translocation may occur independently of the canonical cGAS-STING pathway.

**Figure 1:**
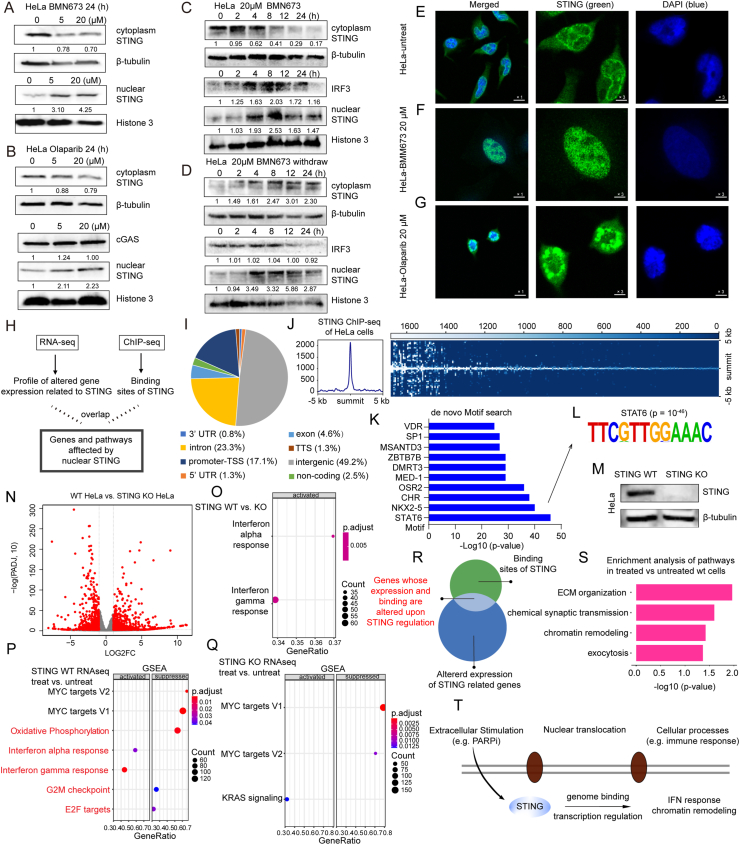
DNA damage promotes the nuclear translocation of STING and activates signaling pathways associated with the innate immune response and chromatin remodeling. **(A)** Western blotting analysis of cytoplasmic and nuclear STING levels in HeLa cells treated with BMN673 for 24 h. **(B)** Western blotting analysis of STING and cGAS levels in HeLa cells treated with olaparib for 24 h. **(C)** Western blotting analysis of STING and IRF3 levels at multiple time points (0, 2, 4, 8, 12, 24 h) following 20 μM BMN673 treatment. **(D)** Western blotting analysis of STING and IRF3 levels at various time points after withdrawal of 24-h treatment with 20 μM BMN673. **(E)** Immunofluorescence staining of STING in untreated HeLa cells. Scale bar: 1 × represents 10 μm. **(F)** Immunofluorescence staining of STING in HeLa cells in response to 20 μM BMN673 treatment for 24 h. Scale bar: 1 × represents 10 μm. **(G)** Immunofluorescence staining of STING in HeLa cells in response to 20 μM olaparib treatment for 24 h. Scale bar: 1 × represents 10 μm. **(H)** Schematic of integrated analysis combining RNA sequencing and chromatin immunoprecipitation sequencing (ChIP-seq). **(I)** Genomic locations of differential STING-binding peaks by Homer annotation. **(J)** The heatmap shows the STING ChIP-seq peak signals in HeLa cells treated with 20 μM BMN673 for 24 h. **(K)** HOMER-identified enriched motifs in STING ChIP-seq data. **(L)** The top motif in STING ChIP-seq of treated HeLa cells is STAT6. **(M)** Western blotting result for STING knockout in HeLa cells. **(N)** The volcano plot shows the differentially expressed genes (DEGs) from STING WT (left) versus STING KO (right) HeLa cells. **(O)** Gene set enrichment analysis (GSEA) showed that the pathways involved in interferon alpha and gamma response were significantly enriched in STING WT cells compared with STING KO HeLa cells. **(P)** GSEA analysis showed the significantly enriched pathways in BMN673-treated cells compared with untreated STING WT HeLa cells. **(Q)** GSEA analysis showed the significantly enriched pathways in BMN673-treated cells compared with untreated STING KO HeLa cells. **(R)** A scheme for identifying genes whose expression and binding are altered upon STING regulation. **(S)** Gene ontology (GO) biological process (BP) analysis was conducted on genes with up-regulated expression in BMN673-treated cells compared with untreated cells within the subset of (R). **(T)** A schematic illustration depicting that extracellular stimulation (*e.g.*, PARPi) initiates nuclear translocation of STING, thereby coordinating a series of cellular processes, including the innate immune response and chromatin remodeling.

Next, we assessed STING protein expression in HeLa cells at various time points (0–24 h) after 20 μM BMN673 treatment (Fig. 1C). Nuclear STING peaked at 8 h, with interferon regulatory factor 3 (IRF3) levels mirroring STING, indicating downstream inflammatory gene activation (Fig. 1C). After BMN673 withdrawal, nuclear STING increased at 4–8 h, with IRF3 remaining elevated within 8 h, suggesting that PARP inhibitors may sustain immune response even after discontinuation (Fig. 1D). The potential rebound of nuclear STING after BMN673 withdrawal may be attributed to two factors: induced STING expression by BMN673, which leads to delayed protein synthesis and subsequent nuclear accumulation, or persistent DNA damage signals that sustain STING's nuclear localization even after BMN673 removal. To investigate whether STING nuclear translocation occurred in other cancer cells and under various DNA damage conditions, we assessed STING expression in MDA-MB-231 breast cancer cells following BMN673 treatment. We found increased nuclear STING and decreased cytoplasmic STING, with cGAS levels unchanged (Fig. S1A and S1B). Additionally, HeLa cells treated with lipopolysaccharide exhibited peak nuclear STING levels at 4–8 h (Fig. S1C–S1E). The nuclear translocation of STING was dose-dependent and independent of cGAS expression.

To confirm STING's cytoplasm-to-nucleus translocation under various DNA damage treatments (BMN673, olaparib, and lipopolysaccharide), we used immunofluorescence in multiple cancer cell lines (HeLa, 769P, B16 cells) (Fig. 1E–G; Fig. S2A–S2F). In all cases, STING was localized to distinct, specific nuclear spots, suggesting roles in specialized cellular processes like gene expression modulation. The degree of STING translocation was also dose-dependent upon olaparib treatment (Fig. 1E–G; Fig. S2A). We therefore hypothesize that nuclear STING interacts with chromatin to participate in the regulation of gene transcription.

To investigate the function of nuclear STING and its regulatory mechanisms, we combined ChIP-seq and RNA-seq data to identify genes involved in STING binding and altered transcription upon STING knockout (Fig. 1H). We used ChIP-seq to map STING binding sites in HeLa cells treated with BMN673 for 24 h, promoting nuclear STING translocation. Our analysis showed that 72% of sites were in introns (23%) and intergenic regions (49%), with only 17% at promoter-transcription start sites (Fig. 1I). Genomic regions within ±5 kb of peak summits had significantly higher binding density (Fig. 1J). The distribution suggests that both intergenic regions and introns harbor a multitude of potential STING binding sites, potentially due to the presence of enhancers within these regions.

To identify key genes and pathways associated with STING binding, we conducted gene ontology (GO) term enrichment analysis. This revealed that many annotated genes were involved in chromatin remodeling and vesicle-mediated transport (Fig. S3A). GO analysis of genes within the promoter-transcription start site region confirmed chromatin remodeling as a major pathway affected by STING (Fig. S3B). Additionally, analysis of transcription factor binding motifs across the human genome identified the most enriched ones, including STAT6, NKX2-5, CHR, and OSR2, which are linked to cancer progression and inflammation (Fig. 1K and L). This suggests that nuclear STING may collaborate with these transcription factors to co-regulate inflammation, chromatin remodeling, and cell fate determination.

Our findings suggest that nuclear STING may regulate gene expression. Next, we engineered a STING knockout (STING KO) HeLa cell line using CRISPR (Fig. 1M) and performed RNA-seq analyses in the presence and absence of DNA damage. Transcriptome analysis of STING KO cells revealed a significant impairment in interferon alpha/gamma response, MHC protein complex assembly, and adaptive immune response pathways compared with wild-type (WT) cells (Fig. 1N and O; Fig. S4A).

To define STING's role in the DNA damage response, we compared transcriptional profiles of STING WT and STING KO cells after 24 h of BMN673 treatment (Fig. 1P and Q; Fig. S5A and S5B). Our analysis revealed a distinct contrast. Gene set enrichment analysis (GSEA) showed that interferon alpha and gamma pathways were activated, while oxidative phosphorylation, G2M checkpoint, and early region 2 binding factor (E2F) target pathways were suppressed only in STING WT cells upon DNA damage, but not in STING KO cells (Fig. 1P and Q). STING acts as a cell-intrinsic metabolic checkpoint, limiting oxidative phosphorylation while also promoting cell death via E2F suppression. This leads to the release of damage-associated molecular patterns (DAMPs) and the recruitment of immune cells, enhancing immune surveillance. In terms of chromatin remodeling, STING influences these pathways by suppressing E2F transcription factors, which are known to stably bind to and regulate the activity of the chromatin-modifying SWI/SNF complex. Furthermore, GO analysis indicated that BMN673 treatment in STING WT cells activated apoptosis, innate immunity, inflammation, and antigen processing pathways (Fig. S5A), whereas in STING KO cells, it affected cell adhesion, angiogenesis, and intermediate filament organization pathways (Fig. S5B). Given the increased nuclear STING protein levels following DNA damage treatment, it is suggested that nuclear STING influences immune and chromatin remodeling pathways, regulating gene expression in these processes.

The complex mechanisms and functions of nuclear STING are still largely unknown. To investigate whether epigenetic and transcriptomic marks jointly regulated gene expression, we integrated ChIP-seq and RNA-seq data to analyze the genes whose expression is altered within STING's genomic binding sites (Fig. 1R). By annotating STING binding peaks from ChIP-seq, we identified 240 genes. We analyzed these genes' expression in our RNA-seq dataset across four conditions: STING WT and STING KO cells with and without BMN673 treatment. Genes up-regulated exclusively in STING WT post-treatment, but not in KO cells, were found to be involved in chromatin remodeling (*e.g.*, REST, ZNF827, RAD54L) and extracellular matrix organization (*e.g.*, MMP24, SMOC1, MATN2) (Fig. 1S). The latter response indicates that STING activation initiates pathways that actively reorganize or remodel the extracellular matrix, potentially contributing to pathological conditions associated with aberrant extracellular matrix deposition or degradation, such as cancer metastasis. These findings reveal nuclear STING's complex functions, suggesting that genes in pathways like chromatin remodeling may be altered in response to DNA damage (Fig. 1T).

Our study highlights the critical role of nuclear STING in the DNA damage response. The findings demonstrate that nuclear STING interacts with chromatin modifiers and immune-related genes, potentially enhancing anti-tumor immunity. Additionally, the activation of nuclear STING could complement the immune responses driven by cytoplasmic STING, thereby potentially strengthening immune-mediated tumor elimination.

## CRediT authorship contribution statement

**Yihao Wang:** Methodology, Conceptualization, Writing – original draft, Formal analysis, Visualization, Data curation. **Zan Shen:** Formal analysis, Writing – review & editing, Methodology. **Lingjie Li:** Validation, Investigation, Supervision, Writing – review & editing, Software. **Jianfeng Shen:** Writing – review & editing, Funding acquisition, Validation, Conceptualization, Supervision.

## Data availability

RNA-seq and ChIP-seq data that support the findings of this study are available in Gene Expression Omnibus (GEO) with accession numbers: GSE296538 and GSE296539. Please contact us to access if it is needed.

## Funding

This study was funded by grants from the General Program of the 10.13039/501100001809National Natural Science Foundation of China (No. 81972667 to J.S.), the National Key Research and Development Program of China (2021YFA1100400 to L.L.), and the Shanghai Municipal Education Commission-Two Hundred Talent (China) (No. 20191817 to J.S.).

## Conflict of interests

There is no conflict of interests in this study.
