# Supplementary material for: DNA damage-induced nuclear STING translocation orchestrates innate immune activation and chromatin remodeling
Source: Genes Dis. 2025 Sep 9;13(3):101851. doi: 10.1016/j.gendis.2025.101851 (PMC12830200; doi:10.1016/j.gendis.2025.101851)
Supplement: Multimedia component 1 [file mmc1.docx]

This file includes:

Supplementary Materials and Methods

Supplementary Figures S1 to S5

**Materials and methods**

Cell culture

HeLa cells (ATCC® CCL-2), 769P cells (ATCC® CRL-1933), B16 cells (ATCC® CRL-6475) were cultured in RPMI-1640 medium (Gibco, #11875093) supplemented with 10% heat-inactivated fetal bovine serum (FBS; Gibco, #10437028) and 1% penicillin-streptomycin (100 U/mL penicillin, 100 µg/mL streptomycin; Gibco, #15140122). MDA-MB-231 cells (ATCC® HTB-26) were cultured in DMEM medium (Gibco, #11965118) supplemented with 10% FBS and 1% penicillin-streptomycin. Cells were maintained in a humidified incubator at 37°C with 5% CO₂.

Nuclear and cytoplasmic extract assay

Chromatin isolation by small-scale biochemical fractionation assay was conducted following the protocol of Wysocka et al: Loss of HCF-1–Chromatin Association Precedes Temperature-Induced Growth Arrest of tsBN67 Cells. Molecular and Cellular Biology (Jun 2001): 3820–3829. Briefly, Harvest 1 × 10^7^ - 2 × 10^7^ cells by using a cell scraper; spin down at 1000 rpm for 2 min, discard supernatant. Wash cell pellet with PBS, spin down at 1000 rpm for 2 min. Repeat the step. Resuspend cell pellet in 200 μl of Buffer A. Add Triton X-100 to a final concentration of 0.1%. Incubate cells on ice for 8 min. Centrifuge at 1,300 x g, 4 ℃, for 5 min; separate supernatant = fraction S1 from pellet (nuclei) = fraction P1. Clarify S1 by high-speed centrifugation at 20,000 x g, 4 ℃, for 5 min; collect supernatant=fraction S2 (discard P2). Wash P1 once with Buffer A and lyse it for 30 min in Buffer B (100 μl). Centrifuge at 1,700 x g, 4 ℃, for 5 min; separate supernatant = fraction S3 from pellet (chromatin) =fraction P3. Wash P3 once with Buffer B and resuspend it either in SDS sample buffer (then boil for 10 min at 70 C and analyze chromatin associated proteins by SDS PAGE/Western Blot.

Western blot

Cells were collected and lysed using radioimmunoprecipitation assay (RIPA) lysis buffer (50 mM Tris-HCl, pH 7.5, 150 mM NaCl, 0.25% sodium deoxycholate, 0.1% nonidet P-40, 0.1% Triton X-100) with the complete proteinase inhibitor cocktail (Roche, Switzerland) for 30 min. After centrifugation at 13,000 g for 15 min at 4 °C, the supernatant was collected and quantified using a BCA assay kit (Solarbio, #PC0020). After the addition of the loading buffer, the cell lysis was denatured for 10 min at 100 °C for the subsequent SDS-PAGE. Then the proteins were transferred to nitrocellulose membranes (Pall, USA). Membranes were blocked with 5% skim milk at room temperature for 2 h, followed by incubation with STING rabbit anti-human antibody (CST, #13647) at 4˚C overnight. After washing the membranes with PBST (PBS, 0.05% Tween-20) 4 times (5 min per wash), the membranes were incubated with the secondary antibody (1:5000) (peroxidase-conjugated goat anti-rabbit IgG, Zsbio, #ZB-2301) at room temperature for 2 h. Finally, membranes were washed with PBST 4 times (5 min per wash). The antibody-reactive bands were revealed by enhanced chemiluminescence (Thermo Fisher, #32209) and exposed on radiographic film.

Immune fluorescence staining

Cells were cultured on sterile coverslips placed in 12-well plates. Cells were seeded at a density of 2×10⁴ cells/mL (1 mL per well) and allowed to adhere for 12 hours under standard culture conditions. After adherence, cells were fixed with 4% paraformaldehyde (PFA) for 15 minutes at room temperature, blocked in 10% Fetal Bovine Serum, 2% Normal Mouse Serum, 0.1% Tween-20 TBS for 1 hour at RT and incubated overnight at 4℃ with STING rabbit anti-human antibody (CST, #13647). Samples were washed and incubated with secondary fluorescently labeled antibody for 1 hours at RT in the dark before nuclear staining with DAPI (ThermoFisher, #D1306). Coverslips were mounted onto slides using ProLong™ Diamond Antifade Mountant (Thermo fisher, #P36962) and imaged using the PerkinElmer Vectra3 multispectral imaging platform. Signal quantification and spectral unmixing were performed with PerkinElmer’s inForm software and the Opal Kit protocol. Final image processing and analysis were conducted using Case Viewer.

RNA-seq analysis

RNA libraries were sequenced using the Illumina NovaSeq 6000 platform. Raw RNA-seq data were trimmed using Trim Galore (v 0.6.6). Alignment by Hisat2 tool (v 2.2.1) was used with default parameters. The generation of read counts for each gene was performed by featureCounts (v 2.0.1). After then the RPKM (Reads per kilo base per million mapped reads) values were computed. Differentially expressed genes (DEGs) were detected using DESeq2 (v 1.26.0) based on absolute log2 transformed fold-change values > 2 and adjusted values of p < 0.05 after applying the Benjamini–Hochberg correction. All R packages were run in version 4.0.3. The GSEA analysis (v 4.2.3) was used to explore the whole-transcriptome dataset.

CRISPR-Cas9 knockout

STING knockout was performed using the CRISPR-Cas9 system. Single-guide RNAs (sgRNAs) targeting exonic regions of STING were designed using the CRISPR Design Tool (crispr.mit.edu) to minimize off-target effects. Two independent sgRNAs per target gene were selected and cloned into the lentiCRISPR v2 plasmid (Addgene, #52961) through Esp3I (BsmBI) restriction sites. Sequences were verified by Sanger sequencing. Human HEK293T/HeLa cells were transfected with sgRNA-Cas9 plasmids using Lipofectamine 3000 (Thermo Fisher), following manufacturer protocols. At 48 hours post-transfection, puromycin (2 μg/mL) was applied for 72 hours to select transfected cells. Surviving cells were expanded as polyclonal populations or single-cell cloned by limiting dilution. Control cells underwent identical procedures using non-targeting sgRNA constructs.

Chromatin Immunoprecipitation Sequencing (ChIP-seq) assay

ChIP-seq was performed to profile genome-wide binding of STING in HeLa cell line treated with 20 μM BMN673 for 24 h. Briefly, treated HeLa cells were cross-linked with 1% formaldehyde for 10 min at room temperature, quenched with 125 mM glycine, and washed with ice-cold PBS. Cells were lysed in SDS lysis buffer (1% SDS, 10 mM EDTA, 50 mM Tris-HCl pH 8.1) supplemented with protease inhibitors, and chromatin was sheared to 200–500 bp fragments using a Covaris S220 sonicator (peak power: 75 W, duty factor: 20%, cycles/burst: 200, duration: 15 min). Sheared chromatin was diluted 10-fold in ChIP dilution buffer (0.01% SDS, 1.1% Triton X-100, 1.2 mM EDTA, 16.7 mM Tris-HCl pH 8.1, 167 mM NaCl) and pre-cleared with Protein G magnetic beads (Thermo Fisher, #88847) for 1 h at 4°C. Immunoprecipitation was performed overnight at 4°C using 10 µl of STING antibody (Abcam, #ab239074). Antibody-chromatin complexes were captured with Protein G beads, washed sequentially with low-salt, high-salt, LiCl, and TE buffers, and eluted in elution buffer (1% SDS, 0.1 M NaHCO3). Cross-links were reversed by incubating at 65°C overnight with 200 mM NaCl. DNA was purified using the QIAquick PCR Purification Kit (Qiagen, #28104), and libraries were prepared with the NEBNext Ultra II DNA Library Prep Kit (NEB, #E7645S) following manufacturer instructions. Libraries were quantified (Qubit, Thermo Fisher Scientific), assessed for fragment size distribution (Bioanalyzer, Agilent Technologies), and sequenced on an Illumina NovaSeq 6000 platform.

Chip-seq analysis

Raw reads were aligned to the reference genome hg19 using Bowtie2 (v2.4.4) with default parameters. Duplicate reads were removed using Picard Tools (v2.27.4), and peaks were called with MACS2 (v2.2.7.1; q-value < 0.05). Homer2 was used for peak annotations and motif finding. Data visualization was performed using deepTools (v2.3) and R (v4.3.1).

Statistical analysis

Statistical analysis was performed using Prism GraphPad Prim 9.0 software. Unpaired two-tailed Student's *t*-test was used to analyze differences between the two groups and a one-way analysis of variance (ANOVA) was used to analyze the differences of multiple groups. All data are expressed as mean ± S.D.

sgRNAs used for CRISPR knockout

| Genes | forward | reverse |
| --- | --- | --- |
| STING sg1 | CACCGGCGGGCCGACCGCATTTGGG | AAACCCCAAATGCGGTCGGCCCGCC |
| STING sg2 | CACCGCATATTACATCGGATATCTG | AAACCAGATATCCGATGTAATATGC |
| Non-targeting gRNA 1 | CACCGCTGAAAAAGGAAGGAGTTGA | AAACTCAACTCCTTCCTTTTTCAGC |
| Non-targeting gRNA 2 | CACCGAAGATGAAAGGAAAGGCGTT | AAACAACGCCTTTCCTTTCATCTTC |


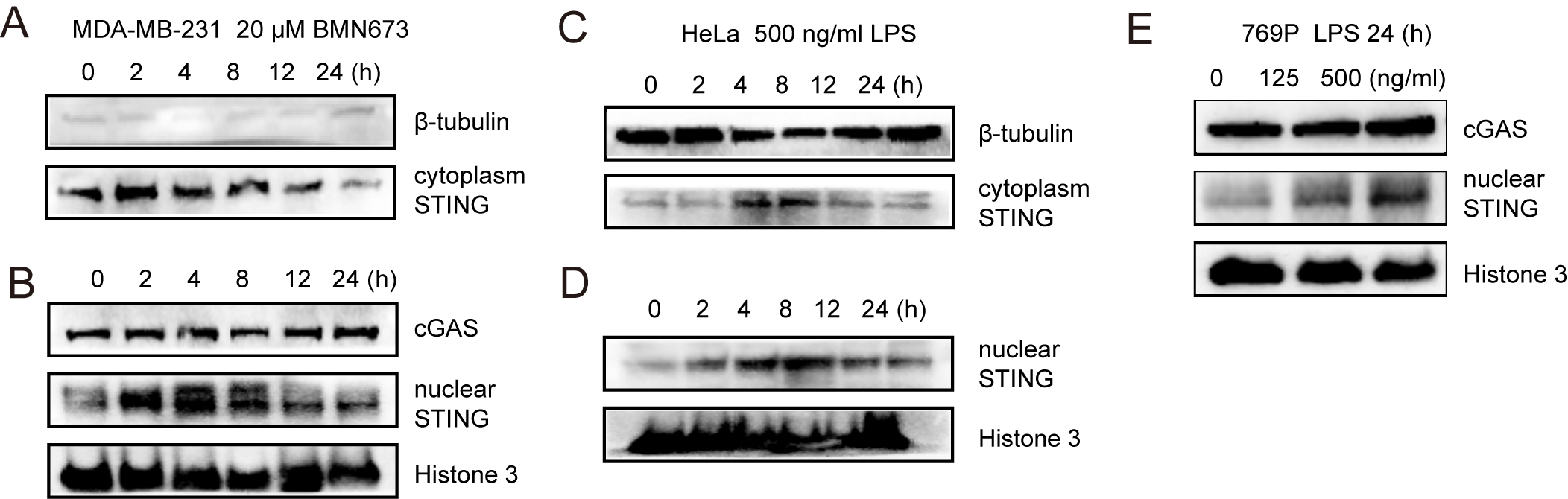
**Figure S1 The translocation of STING to the nucleus promoted by DNA damage was detected by Western blot.**

(A) Western blot analysis of cytoplasm STING of MDA-MB-231 cells in response to 20 μM BMN673 treatment at multiple time points (0, 2, 4, 8, 12, 24 hours). (B) Western blot analysis of nuclear STING and cGAS of MDA-MB-231 cells in response to 20 μM BMN673 treatment at multiple time points. (C) Western blot analysis of cytoplasm STING of HeLa cells in response to 500 ng/ml LPS treatment at multiple time points. (D) Western blot analysis of nuclear STING of HeLa cells in response to 500 ng/ml LPS treatment at multiple time points. (E) Western blot analysis of nuclear STING and cGAS of 769P cells in response to 0, 125 or 500 ng/ml LPS treatment for 24 hours.


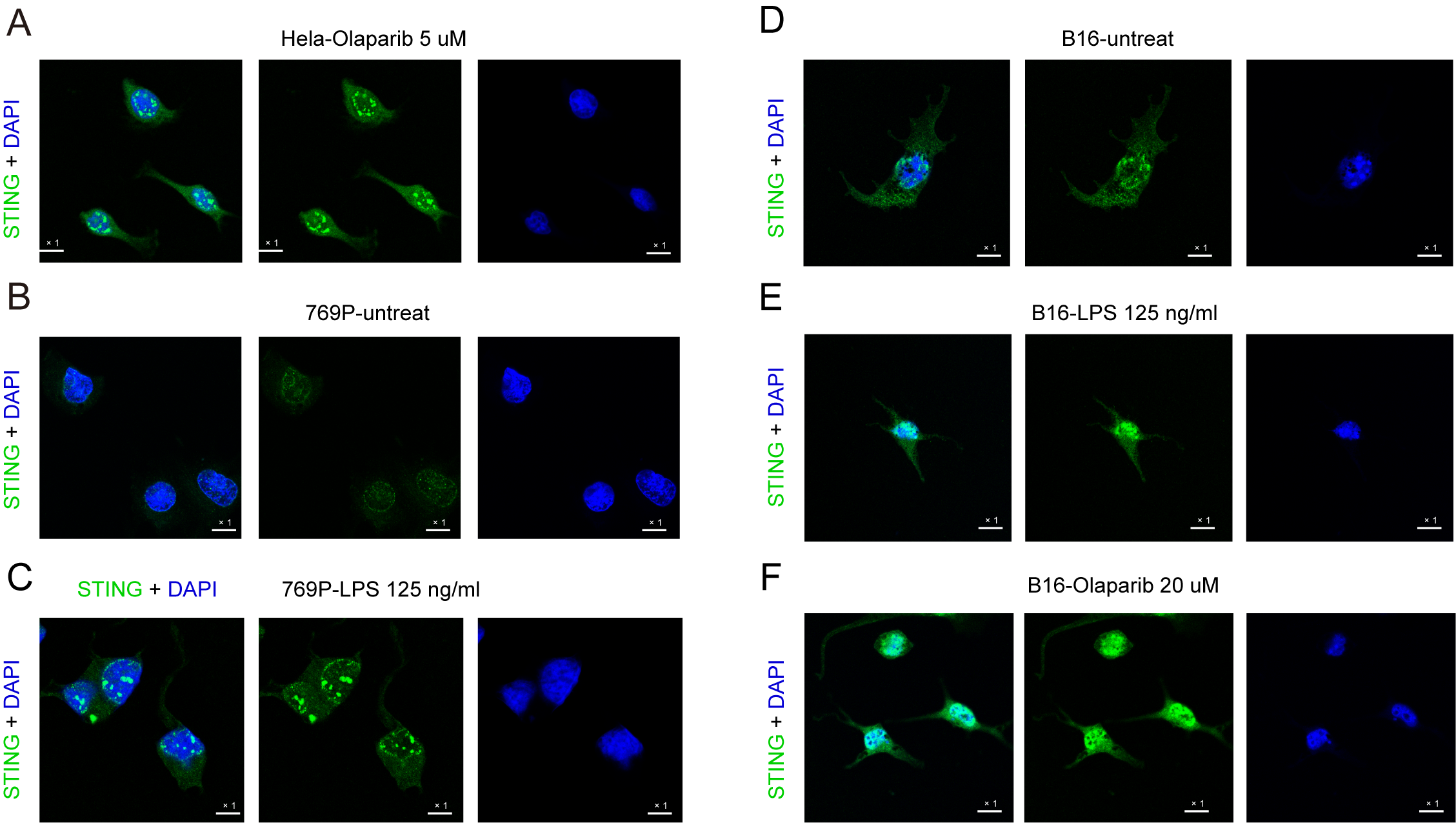


**Figure S2 The translocation of STING to the nucleus promoted by DNA damage was detected by immunofluorescence.**

(A) Immunofluorescence staining of STING in HeLa cells in response to 5 μM olaparib treatment for 24 hours. (B) Immunofluorescence staining of STING in untreated 769P cells. (C) Immunofluorescence staining of STING in 769P cells in response to 125 ng/ml LPS treatment for 24 hours. (D) Immunofluorescence staining of STING in untreated B16 cells. (E) Immunofluorescence staining of STING in B16 cells in response to 125 ng/ml LPS treatment for 24 hours. (F) Immunofluorescence staining of STING in B16 cells in response to 20 μM olaparib treatment for 24 hours. Scale bar: 1× represents 10 μm.


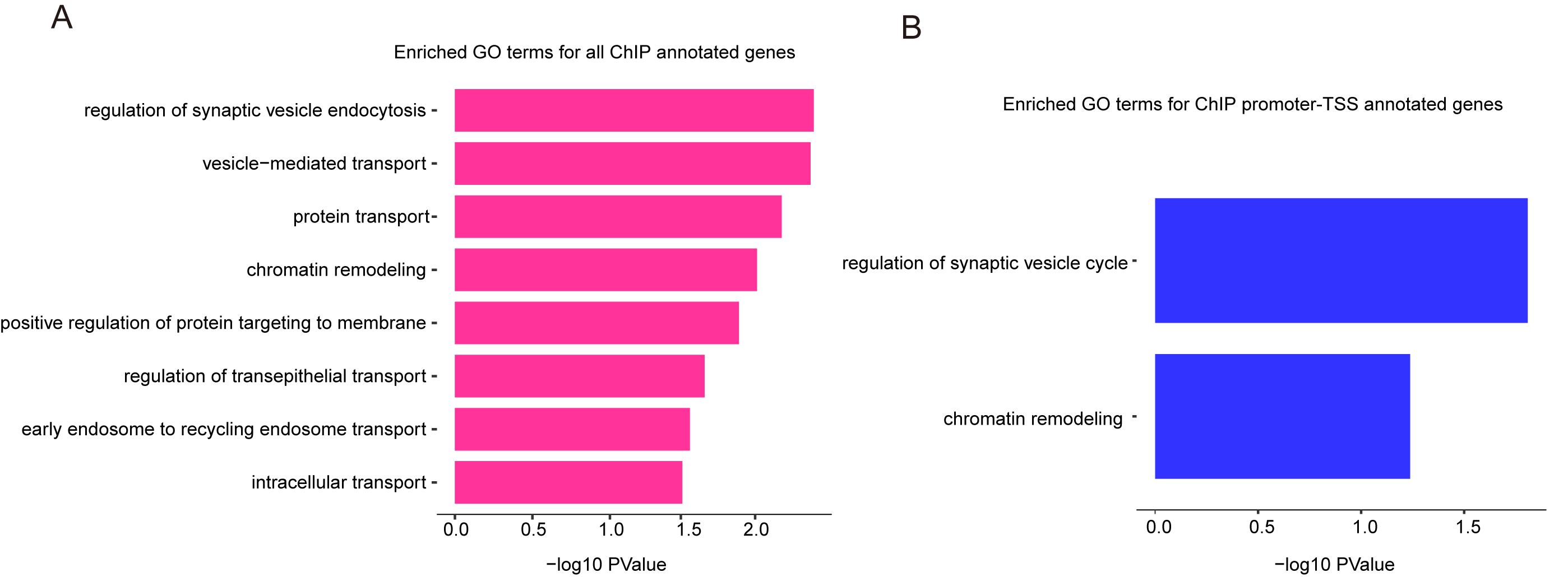


**Figure S3 The STING binding genome regions are associated with pathways including chromatin remodeling.**

(A) Gene ontology (GO) biological process (BP) analysis was performed on genes annotated by Homer 2 using all ChIP-seq peaks to identify their functional categories. (B) GO BP analysis was performed on genes annotated by Homer 2 using ChIP-seq peaks within promoter-TSS regions to identify their functional categories.


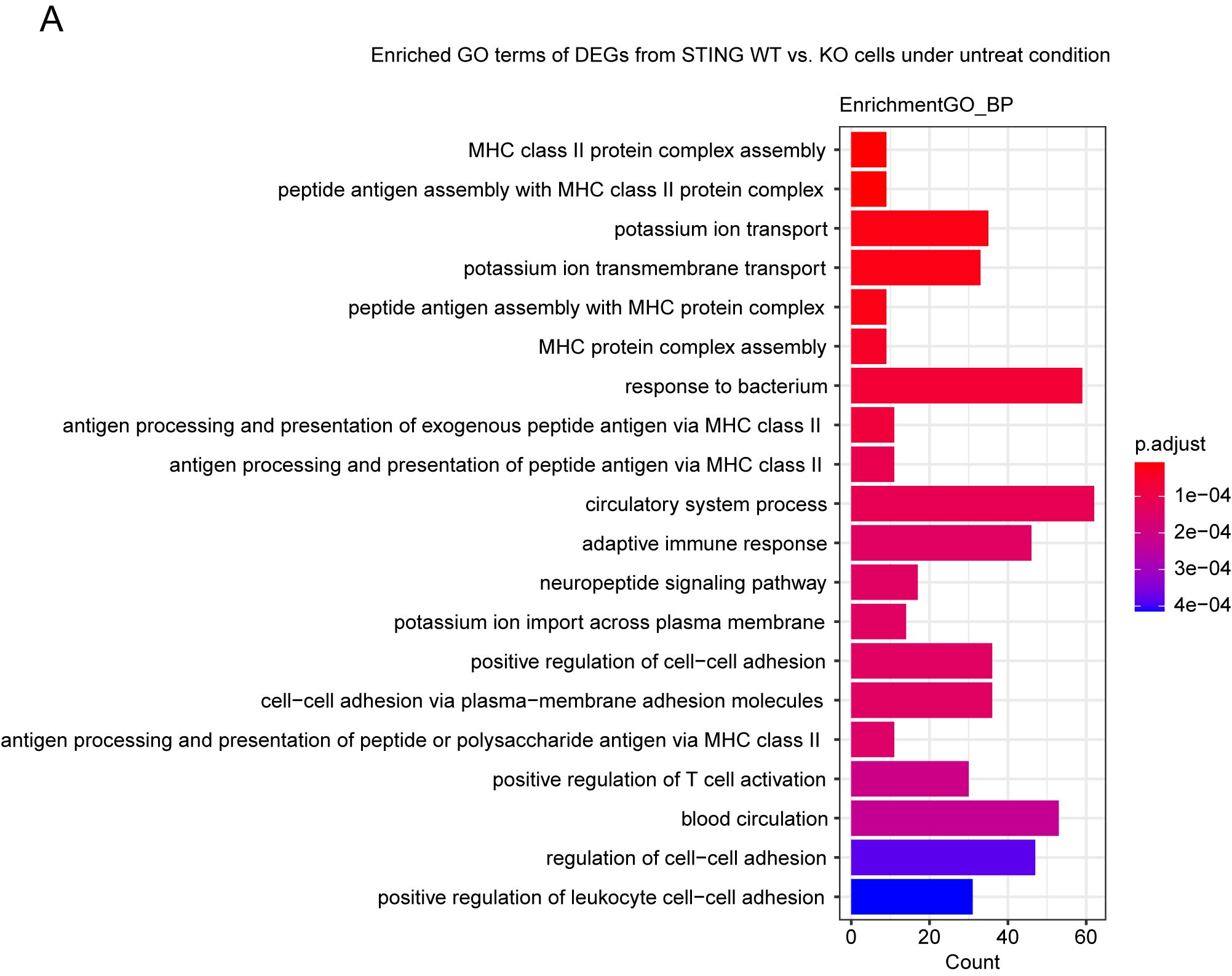


**Figure S4 STING knockout deactivates immune signaling pathways.**

(A) Enrichment analysis of GO BP terms was performed on differentially expressed genes (DEGs) from STING wild-type (wt) versus STING knockout (ko) HeLa cells under untreated conditions.

**
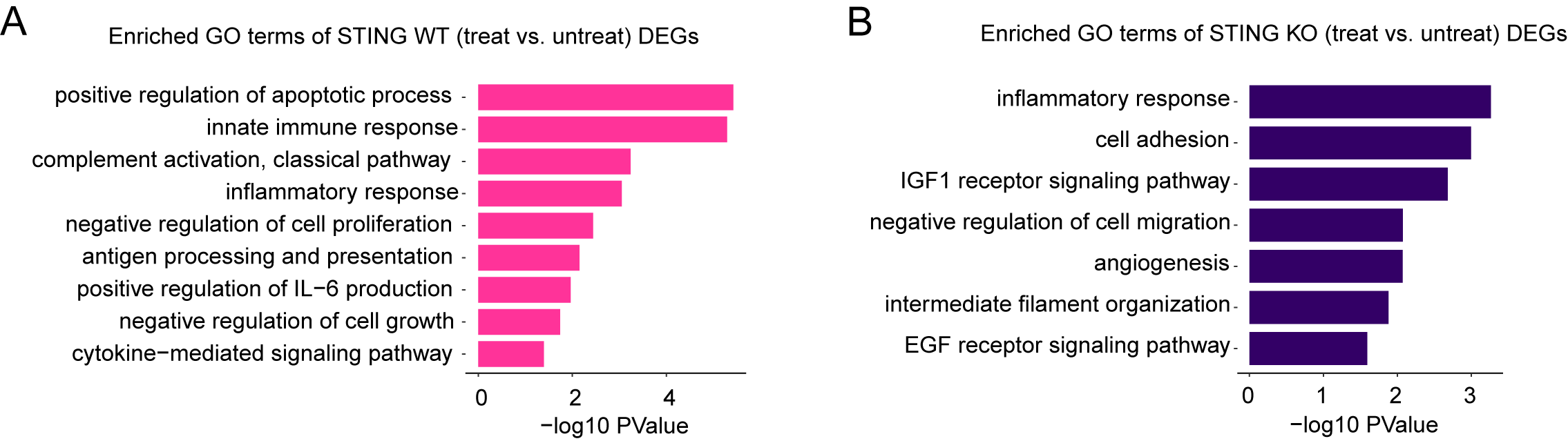
**

**Figure S5 STING facilitates immune response to DNA damage.**

(A) Enrichment analysis of GO BP terms was performed on DEGs from STING wt HeLa cells treated with 20 μM BMN673 for 24 hours, compared to untreated controls. (B) Enrichment analysis of GO BP terms was performed on DEGs from STING ko HeLa cells treated with 20 μM BMN673 for 24 hours, compared to untreated controls.
